# Noxa mitochondrial targeting domain induces necrosis via VDAC2 and mitochondrial catastrophe

**DOI:** 10.1038/s41419-019-1753-4

**Published:** 2019-07-08

**Authors:** Ji-Hye Han, Junghee Park, Seung-Hyun Myung, Sung Hang Lee, Hwa-Young Kim, Kyung Sook Kim, Young-Woo Seo, Tae-Hyoung Kim

**Affiliations:** 1 Department of Biochemistry and Molecular Biology, Chosun University School of Medicine, 309 Pilmoon-Daero, Dong-Gu, Gwang-Ju, 61452 Korea; 2 Department of Molecular and Cellular Biology, Chosun University School of Medicine, 309 Pilmoon-Daero, Dong-Gu, Gwang-Ju, 61452 Korea; 3 Department of Biochemistry and Molecular Biology, Yeungnam University College of Medicine, Daegu, Korea; 4 Department of Biomedical Engineering, College of Medicine, Kyung Hee University, Seoul, Korea; 5 Korea Basic Science Institute Gwang-Ju Center, Chonnam National University, 77, Yongbong-ro, Buk-gu, Gwang-ju, 61186 Korea

**Keywords:** Peptides, Cell death

## Abstract

Noxa, a Bcl-2 homology 3 (BH3)-only protein of the Bcl-2 family, is responsive to cell stresses and triggers apoptosis by binding the prosurvival Bcl-2-like proteins Mcl1, Bcl_XL_, and Bcl2A1. Although the Noxa BH3 domain is necessary to induce apoptosis, the mitochondrial targeting domain (MTD) of Noxa functions as a pronecrotic domain, an inducer of mitochondrial fragmentation, and delivery to mitochondria. In this study, we demonstrate that the extended MTD (eMTD) peptide induces necrotic cell death by interaction with the VDAC2 protein. The eMTD peptide penetrates the cell membrane, causing cell membrane blebbing, cytosolic calcium influx, and mitochondrial swelling, fragmentation, and ROS generation. The MTD domain binds VDACs and opens the mitochondrial permeability transition pore (mPTP) in a CypD-independent manner. The opening of mPTP induced by eMTD is inhibited either by down-regulation of VDAC2 or by the VDACs inhibitor DIDS. These results indicate that the MTD domain of Noxa causes mitochondrial damage by opening mPTP through VDACs, especially VDAC2, during necrotic cell death.

## Introduction

As a Bcl-2 homology 3 (BH3)-only protein of the Bcl-2 family, Noxa binds the prosurvival Bcl-2-like proteins Mcl1, Bcl_XL_, and Bcl2A1, and weakly activates apoptosis^1,2^. Noxa also directly binds the proapoptotic protein Bax, although its affinity is significantly lower than that of the other BH3-only proteins Bim or Bid^1,3,4^. Previously, we showed that Noxa has both a BH3 domain and a mitochondrial targeting domain (MTD), and these domains facilitate mitochondrial targeting and apoptosis^5^. Interestingly, mitochondrial fragmentation depends on the MTD, but not the BH3 domain^6^. Furthermore, MTD peptides conjugated with cell-penetrating peptides (CPP) like eight arginines triggered necrotic death in various cells by opening the mitochondrial permeability transition pore (mPTP) and inducing mitochondrial calcium efflux^7,8^.

Voltage-dependent anion channels (VDACs) are the most abundant protein of the mitochondrial outer membrane (OMM). They play many important roles as channels for transporting metabolite and ions on the mitochondria, regulators of mPTP, and mediators for apoptosis. There are three isoforms of VDACs (VDAC1, 2, and 3) of human and the distinct function is still elucidate. While VDAC1 and 2 are strong pore-forming proteins, VDAC3 acts as a regulatory protein rather than a channel^9^. The little is known about distinct functions of VDAC1 and 2 associated with mPTP, although VDAC1 showed proapoptotic effects interacting with Bax and VDAC2 showed pivotal effects interacting with Bak^10–13^.

Here, we investigated the molecular mechanism by which MTD activates mPTP opening and necrotic cell death. We found that MTD binds VDAC2 and activates mPTP opening in a cyclophilin D (CypD)-independent manner. In addition, extended MTD (eMTD) peptides without CPP induced necrotic cell death by opening the mPTP, causing mitochondrial fragmentation and cytoplasmic membrane damage. These results indicate that MTD is a key Noxa region involved in mitochondrial damage during the process of cell death.

## Results

### eMTD∆4 derived from Noxa induces necrosis via cytosolic Ca^2+^ influx

**Fig. 1:**
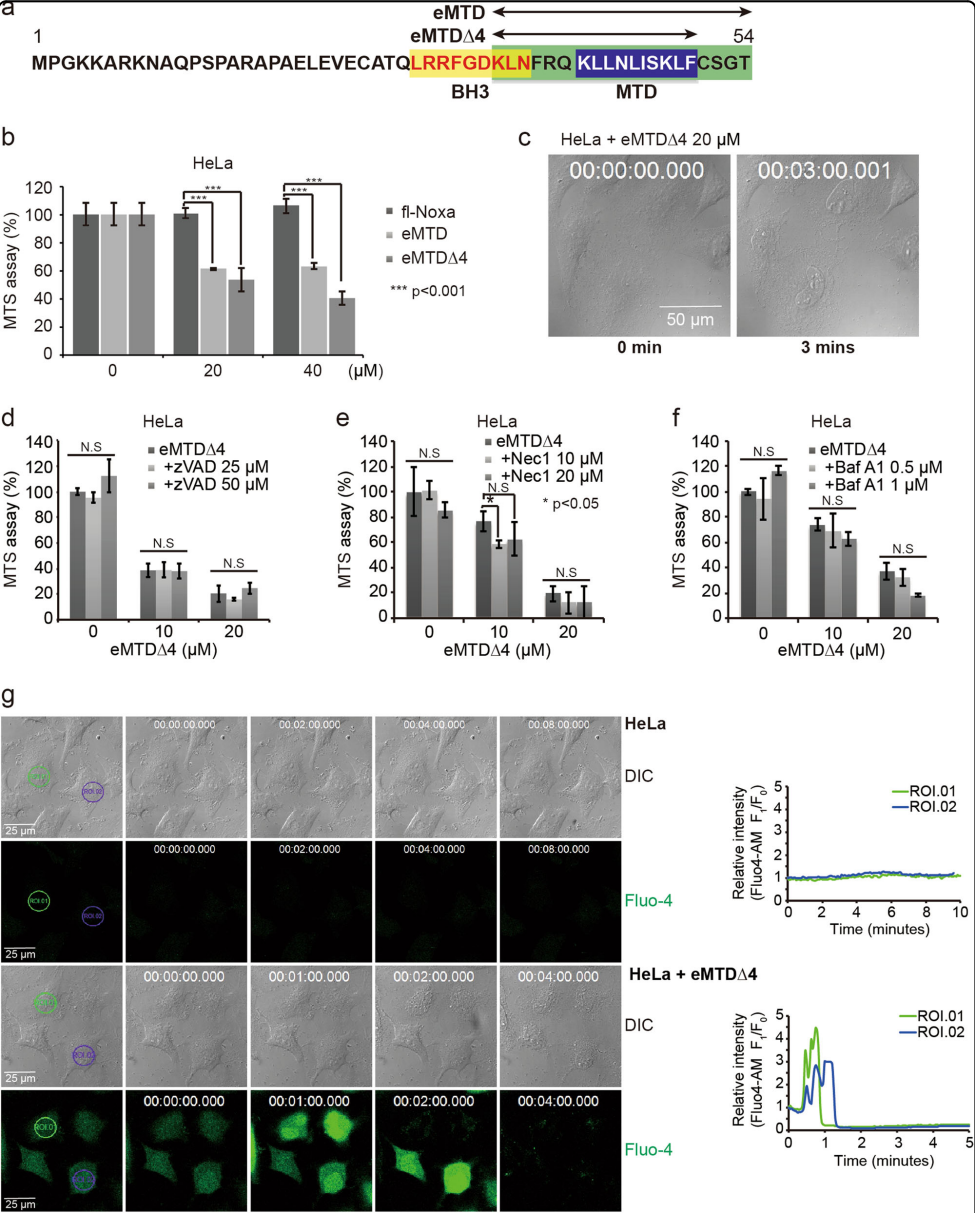
eMTDΔ4 of NOXA induces necrotic cell death via cytosolic Ca^2+^ influx. **a** Noxa domains such as MTD, BH3, eMTD, and eMTDΔ4 are indicated. **b** HeLa cells were treated with full-length (fl) recombinant Noxa protein (20 μM), eMTD (20 μM), and eMTDΔ4 (20 μM), and cell viability was measured by MTS assay. **c** Images of HeLa cells treated with eMTDΔ4 were obtained using bright-field microscopy. **d**–**f** HeLa cells were treated with eMTDΔ4 in the presence of zVAD-fmk (**d**), Necrostatin 1 (**e**), and Bafilomycin A1 (**f**). **g** Images of HeLa cells with or without eMTDΔ4 were captured by time-lapse confocal microscopy in HBSS buffer containing Ca^2+^. Cytosolic Ca^2+^ concentration was visualized using Fluo-4-AM. The relative fluorescent intensity of Fluo-4-AM in two regions of interest (ROI) was graphed over time. All results are represented as means and standard deviation from triplicate samples. *p < 0.05, **p < 0.005, and ***p < 0.001, samples versus control

MTD containing 10 amino acids (41–50 a.a. of Noxa) without CPP did not induce necrosis or mPTP opening in isolated mitochondria, whereas R8:MTD induced necrosis in various cells^7^. These facts suggested that MTD is necessary, but not sufficient for the induction of necrosis or for mPTP opening. Thus, we designed new peptides that contain MTD and its flanking region, as indicated in Fig. 1a. While full-length Noxa (Fig. 1b) and MTD peptides (data not shown) showed no cytotoxic effects, the extended MTD (eMTD, 35–54 a.a. of Noxa), interestingly, had a considerable lethal effect on HeLa cells. Moreover, the shorter extended MTD peptide (eMTD∆4, 35–50 a.a. of Noxa) exhibited the strongest cell killing effect (Fig. 1b, Extended Data Fig. 1a); thus, we used eMTD∆4 instead of eMTD in subsequent studies. The morphological changes in eMTD∆4-treated HeLa cells were observed within 5 min after treatment (Fig. 1c). Cell membrane blebs were quickly formed and burst, implicating that necrosis rather than apoptosis was the type of cell death induced by eMTD∆4. Moreover, eMTD∆4-induced cell death was not inhibited by typical cell death inhibitors such as zVAD-fmk for apoptosis (Fig. 1d), Necrostatin-1, GSK 872 for necroptosis (Fig. 1e, Extended Data Fig. 1b), and Bafilomycin A1 for autophagy (Fig. 1f).

Previously, we reported that MTD with CPP induced mPTP opening and cytosolic Ca^2+^ influx^7^. We expected that eMTD∆4 would also induce cytosolic Ca^2+^ influx. Indeed, eMTD∆4-treated HeLa cells exhibited cytosolic Ca^2+^ influx, which was observed using a Ca^2+^ indicator (Fluo-4) (Fig. 1g). Fluo-4 intensities increased within 1–2 min after treatment with eMTD∆4, and several small crenate patterns of cytosolic Ca^2+^ were observed in cytosolic Ca^2+^-spiked cells. After that, a few big blebs appeared on the cell membrane, the cytosolic Ca^2+^ level decreased drastically, and then the blebs burst. Finally, the cytosolic Ca^2+^ level (Fluo-4 intensity) almost vanished (Fig. 1g).

Calcium entering the cytosol in response to eMTD∆4 can be inferred to originate from intracellular organelles, especially the ER and mitochondria, or from extracellular space. The cytosolic Ca^2+^ spike in response to eMTD∆4 might be originated from intracellular organelles, since it was observed in the buffer with or without Ca^2+^. Fluo-4 intensities showed two kinds of patterns, such as a crenate pattern or a sharp pulse-like pattern, depending, respectively, on the presence or absence of Ca^2+^ in the medium (Fig. 1g and Extended Data Fig. 1c). Vanished Fluo-4 and collapsed cell membrane integrity indicated the leakage of cytosol contents. There was little or no difference in the cytotoxicity of eMTD∆4 in the presence or absence of Ca^2+^ in the buffer. The latter indicates that the Ca^2+^ from intracellular organelles is sufficient to cause cell death by eMTD∆4. Together with our previous results for R8:MTD in that the cytosolic Ca^2+^ was originated from the mitochondria in the Ca^2+^-omitted buffer^7^, these results suggest that mitochondria might play a key role in eMTD∆4-induced cell death.

### eMTD∆4 of Noxa induces mitochondrial swelling, fragmentation, mPTP opening, and ROS generation

**Fig. 2:**
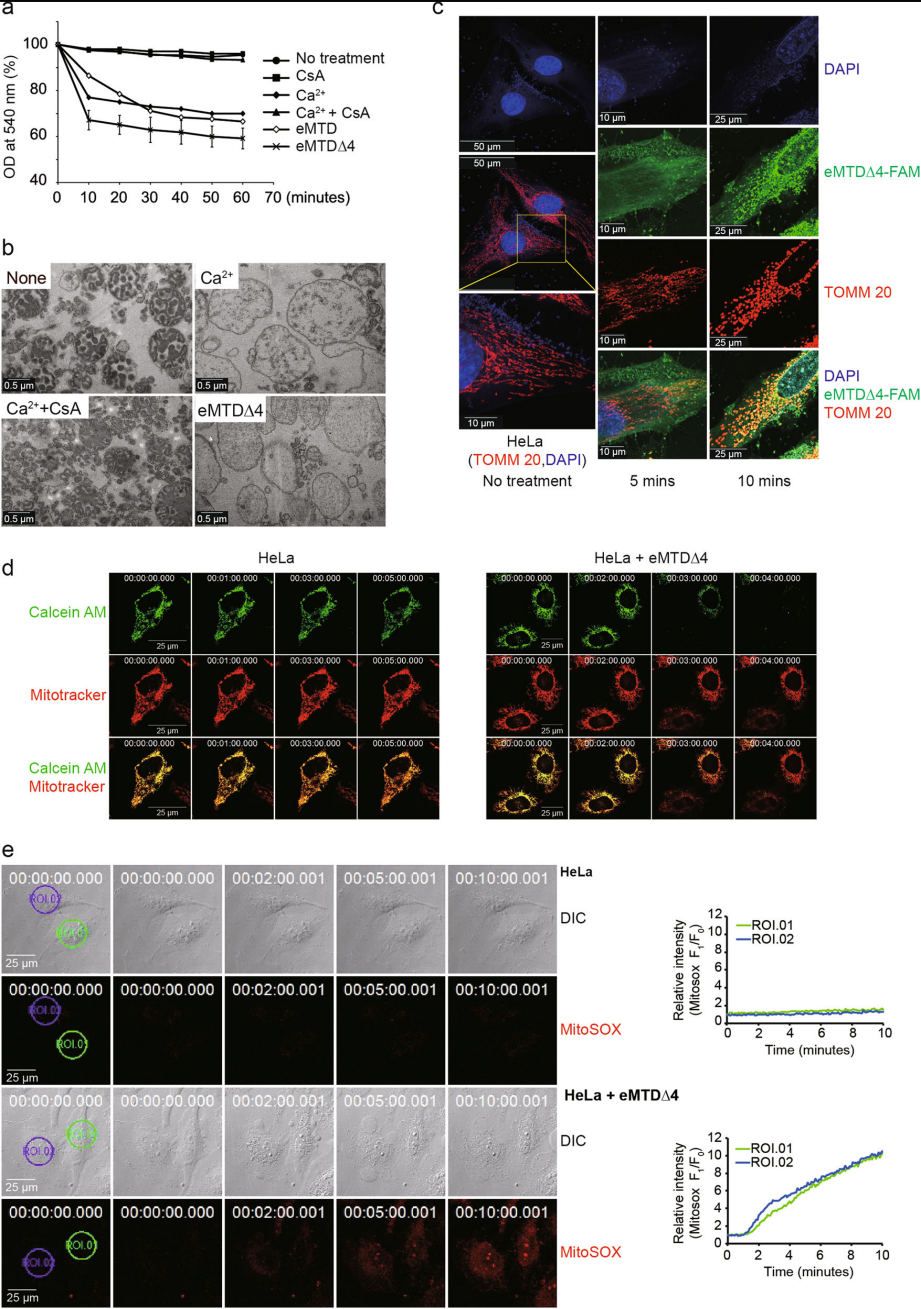
eMTDΔ4 of NOXA induces mitochondrial swelling, fragmentation, PT opening, and ROS formation. **a** Isolated mitochondria were treated with Ca^2+^ (200 μM) alone, CsA (20 μM) alone, Ca^2+^ (200 μM) plus CsA (20 μM), eMTD (25 μM), and eMTDΔ4 (25 μM), and the optical density was measured at 540 nm (OD 540). The results are represented as means and standard deviation from triplicate samples. **b** Isolated mitochondria were treated with Ca^2+^ (200 μM) alone, Ca^2+^ (200 μM) plus CsA (20 μM), and eMTDΔ4 (25 μM). The images were taken with transmission electron microscopy. **c** HeLa cells were treated with fluorescein (FAM)-tagged eMTDΔ4 (Green) for indicated time. Mitochondria and nuclei were visualized using anti-TOMM20 antibody (Red) and DAPI (Blue), respectively. **d** HeLa cells were pretreated with Calecin-AM plus cobalt and Mitotracker to visualize mitochondrial calcium and mitochondria, respectively. The cells were treated with eMTDΔ4 (20 μM). The images were obtained using time-lapse confocal microscope. **e** HeLa cells were pretreated with mitochondrial ROS indicator, MitoSOX, and were treated with eMTDΔ4 (20 μM). The red fluorescence was monitored using time-lapse confocal microscope. The relative fluorescent intensity of MitoSOX in two regions of interest (ROI) was graphed over time

To investigate the direct effects of eMTD∆4 on mitochondria, mitochondrial swelling was assessed by measuring the changes in optical density at 540 nm^14^ and by transmission electron microscopy (TEM, Fig. 2b). Mitochondrial swelling was observed in 10 min after eMTD and eMTD∆4 treatment; the magnitude of mitochondrial swelling induced by eMTD∆4 and eMTD was higher than that induced by Ca^2+^ (Fig. 2a). Although CsA inhibited Ca^2+^-induced mitochondrial swelling as previously reported^15^, CsA had no inhibitory effects on eMTD∆4-induced mitochondrial swelling (Fig. 4c). Fluorescein-tagged eMTD∆4 (eMTD∆4-FAM) showed that eMTD∆4 was colocalized on mitochondria that visualized with anti-TOMM20 (translocase of outer mitochondrial membrane 20) antibody as a mitochondrial indicator, and induced mitochondrial fragmentation (Fig. 2c).

mPTP opening was also observed using calcein-AM and cobalt (a quencher of calcein fluorescence). HeLa cells loaded with calcein-AM and cobalt stained only mitochondria, since cobalt ions quenched calcein-AM in cytosolic and nuclear compartments. When mPTP opens, calcein-AM leaks out to the cytosol from the mitochondria, cobalt ion flows into the mitochondria, and calcein fluorescence decreases^16^. After eMTD∆4 treatment, calcein fluorescence decreased abruptly, indicating that the mPTP was opened by eMTD∆4 (Fig. 2d).

In addition, mitochondrial reactive oxygen species (ROS) were observed using the mitochondrial superoxide indicator, MitoSOX. Several large blebs appeared about 2 min after eMTD∆4 treatment, and the intensity of MitoSOX started to increase afterward, followed by the collapse of the cell membrane (Fig. 2e). Together, eMTD∆4 directly triggered a catastrophe of mitochondria, including swelling, fragmentation, mPTP opening, and ROS generation. These results demonstrate that mitochondria play a critical role in eMTD∆4-induced cell death.

### MTD of Noxa binds VDAC2

To investigate the mechanism by which MTD targets mitochondria, MTD-binding partners were searched using a streptavidin-biotin precipitation method. Biotinylated MTD peptide was incubated with HeLa cell lysates, followed by precipitation with streptavidin-agarose bead, SDS-PAGE, silver staining, and protein sequence analysis using MicroQ-TOF III mass spectrometer (sequence data in Extended Data Fig. 2). Among the candidate proteins identified, voltage-dependent anion-selective channel protein 2 (VDAC2) was considered the most likely candidate because it is embedded in the OMM and contributes to the formation of the mPTP^10,17^.

**Fig. 3:**
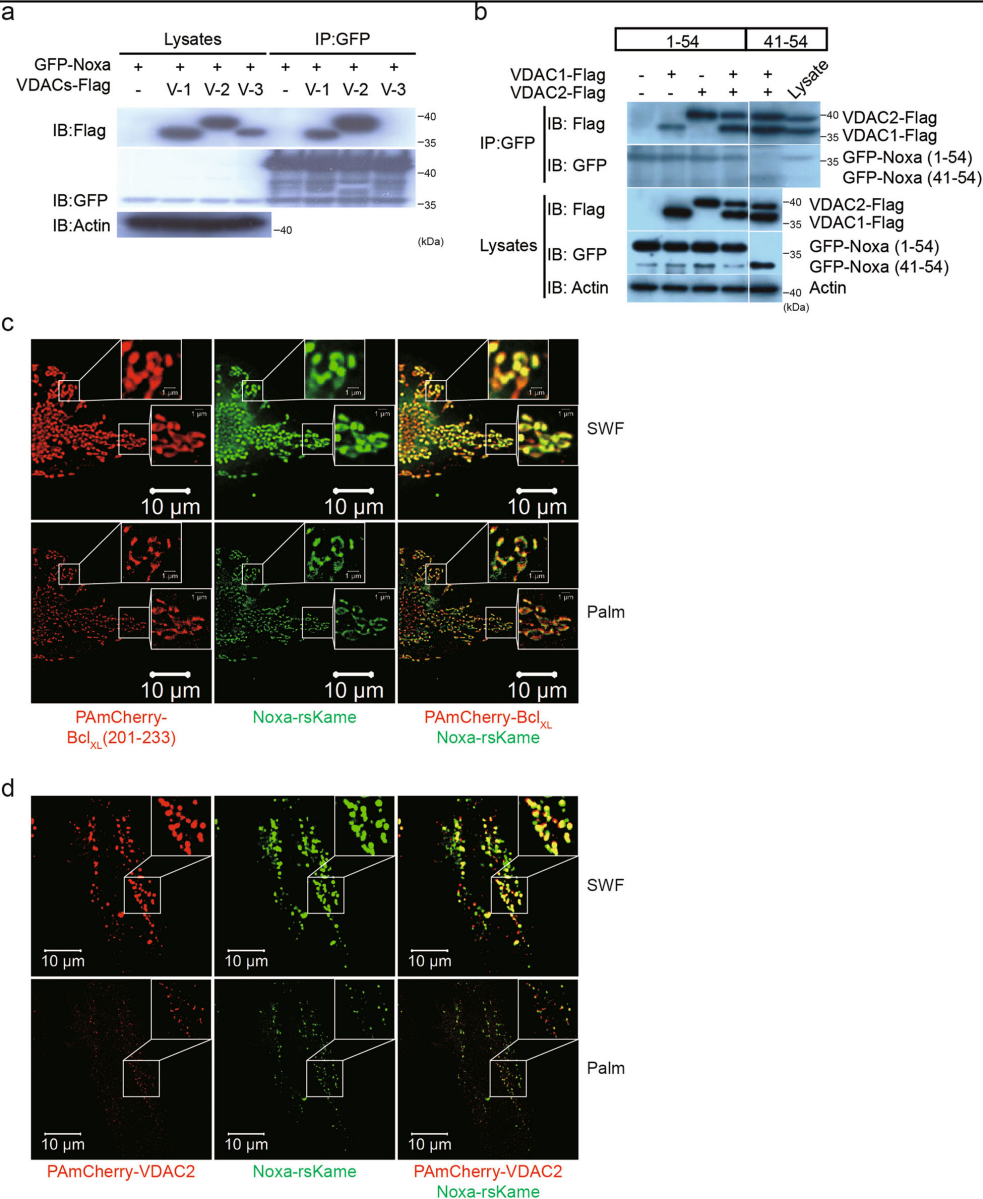
MTD of NOXA binds VDAC2 protein. **a** 293 HEK cells were cotransfected with GFP-Noxa (1-54) and VDAC1, 2, or 3-Flag expression plasmid vectors, and immunoprecipitation assay was carried out using anti-GFP antibody. Immunoblots were done using anti-Flag, anti-GFP, and anti-actin antibodies. **b** The same experiments as described in Fig. 3a were carried out except for cotransfection of expression vectors GFP-Noxa (1-54) or GFP-Noxa (41-54) plus VDAC1 or 2-Flag. **c**, **d** HeLa cells were cotransfected with PAmCherry-Bcl_XL_ (201-233) or (and) Noxa-rsKame (**c**), and PAmCherry-VDAC2 or (and) Noxa-rsKame (**d**). The images for Noxa-rsKame (green), PAmCherry-Bcl_XL_ (201–233) (the mitochondrial outer membrane marker; red), and PAmCherry-VDAC2 (red) were obtained by PALM and SWF. (SWF) summed widefield total internal reflection microscopy (TIRF) (total internal reflection microscope)

To confirm the interaction between Noxa and VDAC2, immunoprecipitation analysis was performed using GFP-Noxa and VDACs overexpression. VDAC1 and VDAC2 bind to GFP-Noxa, unlike VDAC3 (Fig. 3a), and the Noxa region 41–54, which includes MTD, is sufficient to interact with VDAC1/2 (Fig. 3b). The intracellular distribution of Noxa and VDAC2 further support the interaction between Noxa and VDAC2 in HeLa cells observed by photo-activated localization microscopy (PALM). Noxa-rsKame (green color) was codistributed with PAmCherry-Bcl_XL_ (201–233) (red color), an indicator marker of the OMM (Fig. 3c), indicating that Noxa is localized on the OMM. PAmCherry-VDAC2 (red color) showed the same localization pattern as Noxa-rsKame (Fig. 3d), suggesting that VDAC2 is colocalized with Noxa. However, the distribution patterns of Noxa-rsKame in cells coexpressing PAmCherry-Bcl_XL_ (201–233) were different from those in cells coexpressing PAmCherry-VDAC2. This might be due to the oligomerization of PAmCherry-VDAC2, since activated VDAC2 physiologically exists as oligomers^18^. Overexpression of PAmCherry-VDAC2 alone or both PAmCherry-VDAC2 and Noxa-rsKame together may cause oligomerization of PAmCherry-VDAC2, which may alter the distribution pattern of PAmCherry-VDAC2. Despite the oligomerized pattern of VDAC2, the distribution of Noxa overlaps with that of VDAC2, which favors the intracellular interaction between them.

### VDAC2 plays a key role in mPTP opening and necrosis induced by eMTD∆4

**Fig. 4:**
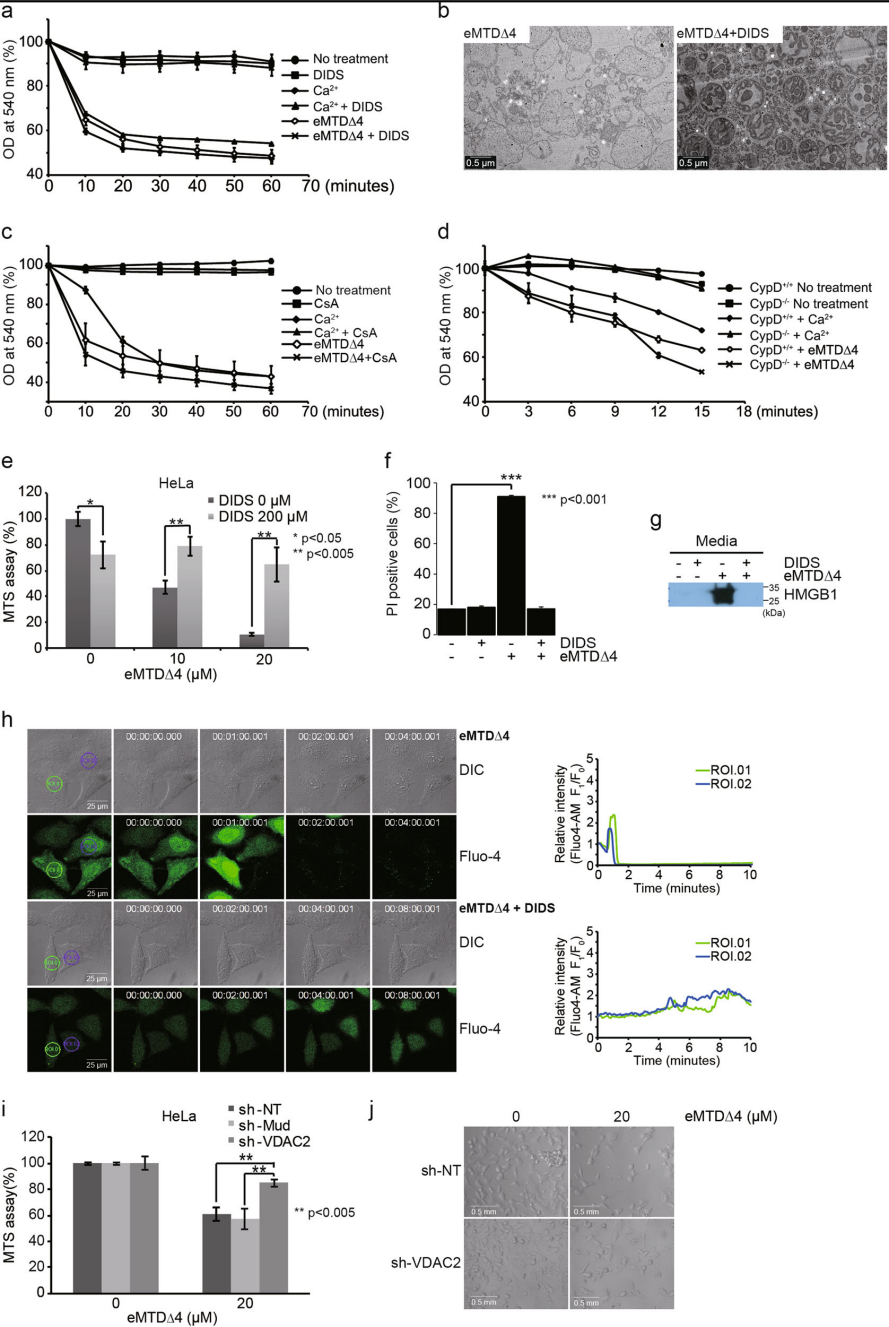
VDAC2 plays an important role in necrotic cell death induced by eMTD∆4. **a**, **c** Isolated mitochondria from BalB/c mouse liver were treated with DIDS (100 μM), Ca^2+^ (200 μM), Ca^2+^ plus DIDS, eMTDΔ4 (25 μM), and eMTDΔ4 (25 μM) plus DIDS (**a**), and were treated with CsA (20 μM), Ca^2+^, Ca^2+^ plus CsA, eMTDΔ4, and eMTDΔ4 plus CsA (**c**). OD at 540 nm was monitored. **b** Isolated mitochondria from BalB/C mice were treated with eMTDΔ4 (25 μM) and eMTDΔ4 plus DIDS. The images were obtained by TEM. **d** Isolated mitochondria from cyclophlin D deficient mice or normal mice were treated with Ca^2+^ and eMTDΔ4. **e**–**f** HeLa cells were treated with DIDS and eMTDΔ4. Cell viability was determined by the MTS assay (**e**), and PI-positive cells (**f**). **g** HeLa cells were treated with DIDS, eMTDΔ4, and eMTDΔ4 plus DIDS, and the media were collected and precipitated for detection of HMGB1 release. **h** Cytosolic Ca^2+^ concentration was visualized using Fluo-4 in HeLa cells treated with eMTDΔ4 and eMTDΔ4 plus DIDS. The relative fluorescent intensity of Fluo-4-AM in two regions of interest (ROI) was graphed over time. **i** and **j**, HeLa cells were transfected with shRNA expression vectors (sh-NT for no target gene, sh-Mud for MUDENG, sh-VDAC2 for VDAC2), and were treated with eMTDΔ4. Cell viability was determined by the MTS assay (**i**), and the images were obtained by bright-field microscopy (**j**). All results are represented as means and standard deviation from triplicate samples. *p < 0.05, **p < 0.005, and ***p < 0.001, samples versus control

To verify the functional relevance of VDAC2 and MTD, mitochondrial swelling was monitored using isolated mitochondria treated with eMTD∆4 in the presence or absence of DIDS. DIDS inhibits VDAC2 oligomerization induced by proapoptotic stimuli^19,20^. As expected, the eMTD∆4-induced mitochondrial swelling was inhibited by DIDS; however, the Ca^2+^-induced mitochondrial swelling was not inhibited by DIDS (Fig. 4a). The mitochondrial morphology examined by TEM confirmed that DIDS completely blocked eMTD∆4-induced mitochondrial swelling (Fig. 4b). Mitochondrial swelling induced by mPTP opening in Ca^2+^-treated mitochondria can be inhibited by a cyclophilin D (CypD) inhibitor CsA^15^, as shown in Fig. 2a. Interestingly, eMTD∆4-induced mitochondrial swelling was not inhibited by CsA (Fig. 4c). In addition, Ca^2+^-induced mitochondria swelling was defective in mitochondria isolated from CypD-deficient mice^21^. However, eMTD∆4 induced mitochondrial swelling in CypD-deficient mitochondria (Fig. 4d). Moreover, the cell killing activity of eMTD∆4 was inhibited by DIDS (Fig. 4e), and PI-stained cell number and HMGB1 release induced by eMTD∆4 in HeLa cells were inhibited by DIDS (Fig. 4f, g), further supporting the evidence that eMTD∆4 induces necrosis via an interaction with VDAC2 that can be inhibited by DIDS. In addition, eMTD∆4-induced calcium spikes in the cytosol were significantly inhibited by DIDS (Fig. 4h). The killing activity of eMTD∆4 in HeLa cells was inhibited by shVDAC2 but not by shMud (an unrelated MUDENG gene) or shNT (no target genes) (Fig. 4i, j, and Extended Data Fig. 4), confirming that VDAC2 plays a key role in eMTD∆4-induced necrosis. Together, these results demonstrate that eMTD∆4 induces mitochondrial swelling by opening the mPTP via VDAC2, which is not involved in the well-known Ca^2+^-induced mPTP opening.

### eMTD∆4 perforates cell membrane

**Fig. 5:**
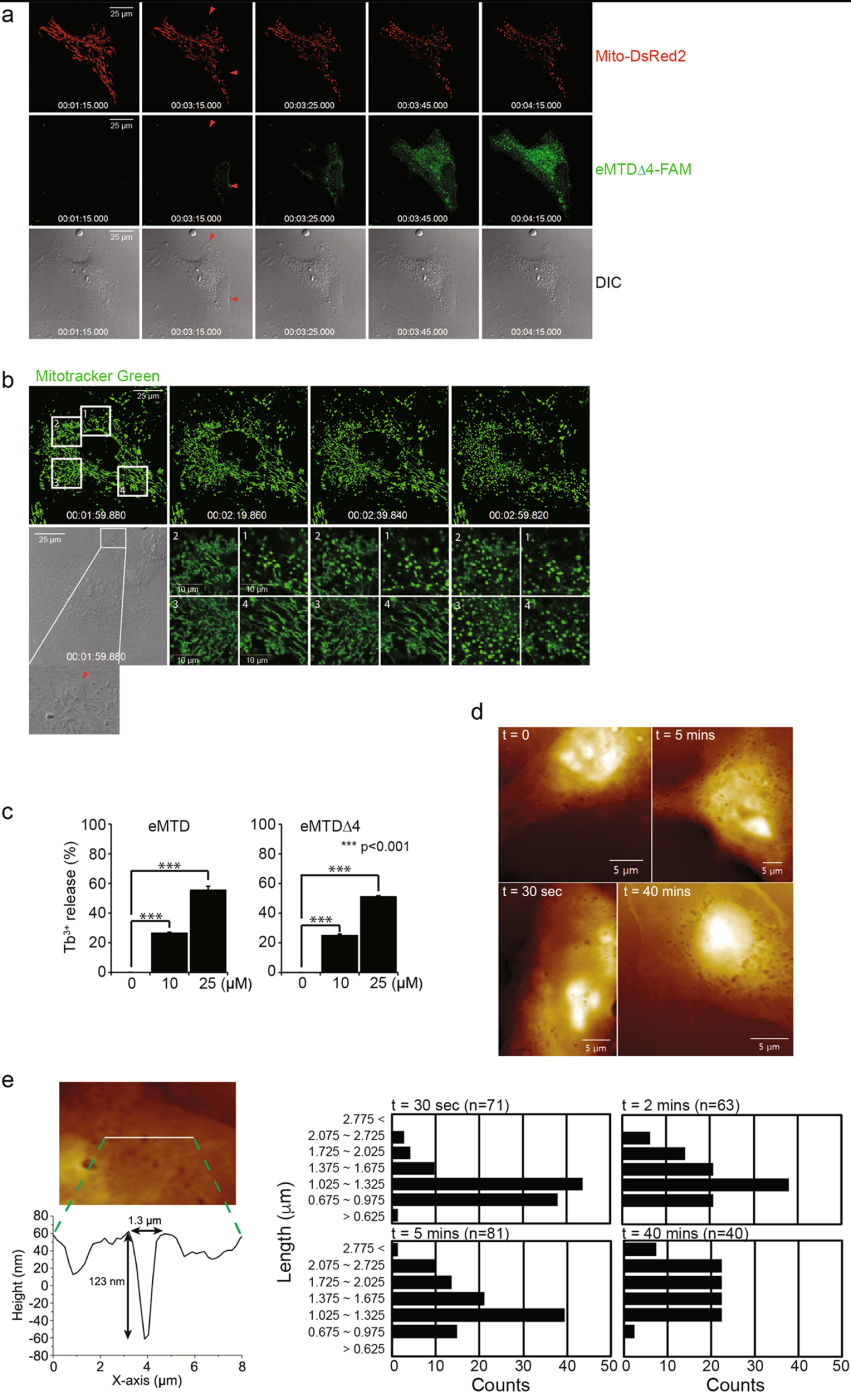
eMTDΔ4 perforates cell membrane. **a** HeLa cells were transfected with Mito-DsRed2 to visualize mitochondria, and were treated with eMTDΔ4-FAM. The images were taken by time-lapse confocal microscopy. **b** HeLa cells were stained with MitoTracker green, and were treated with eMTDΔ4. The images were taken by time-lapse confocal microscopy. **c** Liposome loaded with Tb^3+^ was treated with eMTD and eMTDΔ4, and the fluorescence was measured by the two-step assay. All results are represented as means and standard deviation from triplicate samples. *p < 0.05, **p < 0.005, and ***p < 0.001, samples versus control. **d**, **e** MDA-MB-231 cells were treated with eMTDΔ4 and were fixed at the indicated time. The representative images of the cell surface obtained by AFM were shown (**d**). The images were analyzed with SPIP to measure the diameters of holes in cell membrane, and the indicated number of holes were counted (**e**)

To investigate the process by which eMTD∆4 comes to the mitochondria across the cell membrane, HeLa cells treated with eMTD∆4-conjugated FAM (eMTD∆4-FAM) were monitored using time-lapse confocal microscopy. A few minutes after eMTD∆4 treatment, a small number of tiny blebs were formed on the cell membrane; then, eMTD∆4-FAM started to penetrate and diffuse into the cell at the sites of the tiny blebbing (red arrows), while mito-DsRed2-expressing mitochondria were observed to be faded. Moreover, the fading of DsRed2 in the mitochondria appeared to be synchronized with the diffusion path of eMTD∆4-FAM in the cytosol (Fig. 5a). Indeed, mitochondria fragmentation of HeLa cells treated with eMTD∆4 occurred near the site of the first tiny blebs (red arrow) on the cell membrane (site 1), and sequentially spread out to the cytosol (sites 2, 3, and 4) (Fig. 5b). These results indicate that eMTD∆4 penetrates the cell membrane through a tiny bleb, and then diffuses and targets mitochondria, where it causes mitochondrial fragmentation and mPTP opening.

To investigate the possibility that eMTD∆4 leaks out from the liposome, we generated liposomes composed of phosphoserine (PS), phophoethanolamine (PE) and phosphocholine (PC), and loaded terbium (Tb^3+^) into them. The membrane leakage of the liposome was determined by measuring the complex formation of Tb^3+^ with dipicolinic acid (DPA)^22^. Interestingly, eMTD and eMTD∆4 caused membrane leakage (Fig. 5c). This implies that eMTD and eMTD∆4 are able to penetrate lipid layers without any protein receptors.

In addition, several tiny holes on the cell membrane of HeLa cells treated with eMTD∆4 were directly observed by atomic forced microscopy (AFM) (Fig. 5d). The membrane pores diameters generated by eMTD∆4 were in the range of 0.675–1.325 μm within 30 s after eMTD∆4 administration, and they became larger (2.075–2.725 μm) at 40 min after eMTD∆4 administration (Fig. 5e). Together, these results suggest that eMTD∆4 first attenuates the cell membrane integrity, and then it forms small blebs, penetrates into the cytosol, diffuses into mitochondria, binds VDAC2, and opens the mPTP, causing mitochondrial Ca^2+^ to leak into the cytosol, and thus completing the death process.

## Discussion

Noxa is considered a proapoptotic BH3-only protein and BH3 domain is a responsible domain for inducing apoptosis; hence, the proapoptotic BH3 domain of Noxa has been a major focus of attention in the field^23^. However, previous research has evidenced the ability of MTD to induce necrosis^7^. Although we have shown that R8:MTD can induce necrosis via mitochondrial Ca^2+^ release and mPTP opening^24^, MTD alone (without CPP) was not able to induce necrosis and mPTP opening in isolated mitochondria (Extended Data Fig. 3). Here, we showed that eMTD of Noxa induces necrosis, not apoptosis, via VDAC2 binding and mPTP opening.

Noxa is expressed at the basal level in a stress-free condition, but it may increase its expression level through transactivation in various stress conditions such as DNA damage^25^, proteasome inhibitors^26^ or hypoxia^27^. Hypoxic conditions increase Noxa transcription in several tissues and tumors in the HIF-1α-dependent transactivation^28^. Furthermore, ischemia-reperfusion (IR) has been shown to cause tissue damage through necrosis. IR-injured tissues exhibited ROS formation, Ca^2+^ overload, and mPTP opening^29^. Interestingly, DIDS was reported to reduce IR-injured myocardial damage in rats^30^. These studies indicate that necrosis primarily contributes to IR-induced tissue injury, and that VDACs is a key player in IR-induced tissue damage. The findings that Noxa binds to VDAC2 (Fig. 3) and that eMTD of Noxa induces necrosis (Figs. 1 and 2) suggest that Noxa’s MTD domain is responsible for IR-injured tissue damage or possibly eMTD-like cleavage products derived from full-length Noxa may be involved in IR-induced tissue damage. Although we do not have evidence to support that full-ength Noxa is cleaved by a proteinase, we suspect that full-length Noxa might be cleaved by proteinases during apoptosis. This possibility should be thoroughly investigated in future.

VDAC2 is one of the VDAC isoforms (VDAC1, 2, and 3), distinguished by unique 11–12 amino acid extension at the N-terminal end and its cysteine contents^10^. VDACs (VDAC1 and 2) were initially expected to be the pore-forming component of mPTP because of their pore-forming characteristics; however, they were identified as a regulator of the pore by subsequent studies^9^. The isoform-specific function of VDACs with mPTP is almost unknown and this may result from the similarity of sequences. Our experiments also showed that both VDAC2 and VDAC1 bind to Noxa (Fig. 3). Although we could not fully exclude the possibility that VDAC1 may play a role in eMTD-induced necrosis, there are some reasons why we prefer VDAC2 to be associated with cell killing activity of eMTD. First, VDAC2 down regulation by shRNA did affect the toxicity of eMTDΔ4 in HeLa cells (Fig. 4). Second, in spite of relative abundance of VDAC1, we only found VDAC2 by streptavidin-biotin precipitation method with eMTD^31^. Third, although VDAC1 and VDAC2 have similar amino acid sequences, only VDAC1 is distributed to plasma membrane^32^, and our confocal data with eMTD∆4-FAM showed only mitochondrial localization, suggesting that VDAC2 may be a major target site of eMTD. Fourth, VDAC1 is associated with mitochondrial-dependent cell death via mediating cytochrome c; however, cytochrome c release cannot induce cell death this quickly like eMTD^10,13^. These data could show that VDAC2, among the VDACs, has a major role in the cell death by eMTD.

The very rapid response of HeLa cells to eMTD∆4 was a pulse-like or crenation-like cytosolic Ca^2+^ influx (Fig. 1g, Extended Data Fig. 1C). It is worth to note that Ca^2+^ influx occurred before the membrane blebbing. Based on the finding that eMTD∆4 causes small blebs on the cell membrane (Fig. 5c), we speculate that the cytosolic Ca^2+^ spikes might be a feature of the membrane repair process^33^. The pulse-like cytosolic Ca^2+^ spikes were observed in the absence of Ca^2+^ in buffer (Extended Data Fig. 1C), whereas the crenation-like cytosolic Ca^2+^ spikes were observed in the presence of Ca^2+^ in buffer (Fig. 5c). In the absence of Ca^2+^ in the buffer, the first pulse of cytosolic Ca^2+^ may be triggered by the release of Ca^2+^ from the ER^34^ or possibly from the mitochondria^35^, and then the increased cytosolic Ca^2+^ was quickly decreased possibly owing to the reabsorption of cytosolic Ca^2+^ by the ER or the mitochondria^36^. These repeated changes in the levels of cytosolic Ca^2+^ appeared as the second and third pulses of cytosolic Ca^2+^. However, in the presence of cytosolic Ca^2+^ in the buffer, the increased cytosolic Ca^2+^ might exhibit slight fluctuations, possibly due to the Ca^2+^ influx from outside of the cell, which is part of the membrane repair process that induces the crenation-like cytosolic Ca^2+^ spikes^33^.

After several Ca^2+^ spikes, a couple of membrane blebs appeared, the membrane integrity was disrupted, and eMTD∆4 passively diffused into the cytosol (Fig. 5a). Mitochondria, then, started to fragment (Fig. 5b) and produce ROS (Fig. 2e). Finally, the membrane blebs burst, and cytosolic contents started to leak out (Fig. 1g and Extended Data Fig. 5). These events suggest that the major impact on membrane-bleb-bursts might be caused by the mitochondrial damage induced by eMTD∆4. In summary, these results suggest that eMTD∆4 penetrates into the cytosol by forming small blebs, diffusing into the mitochondria, and leaking mitochondrial Ca^2+^ into the cytosol by opening the mPTP via VDAC2 binding, thus completing the death process.

## Materials and methods

### Cell culture

Cells were cultured in DMEM with 10% fetal bovine serum (FBS, Gibco-Thermo Fisher, MA, USA) and 1X penicillin-streptomycin (100 units/mL and 100 μg/mL, respectively, Gibco-Thermo Fisher, MA, USA) at 37 °C

### Peptide synthesis

Peptides were synthesized and purified by high performance liquid chromatography (HPLC) to achieve a purity of over 95% (Anygen, Gwangju, South Korea). The peptides were dissolved in distilled water and stored at −20 °C.

### MTS assay

Cells were cultured on 96-well plates to reach 80–90% confluency. They were then treated with peptides and chemicals in phenol red-free DMEM for an hour (final volume 100 μL). In the case of DIDS treatment, after the incubation with peptides, the media were replaced with fresh media, because of the yellow color of DIDS. MTS solution (20 μL; Promega, WI, USA) was added and the cells were incubated for an hour. The absorbance at 450 nm was measured.

### Confocal microscopy

HeLa cells were cultured on a Lab-Tek Chamber glass slide. Fluo-4-AM (5 μM), MitoSOX (5 μM), and DCF (5 μM) (Thermo Fisher, MA, USA) were added in HBSS buffer (0.49 mM MgCl_2_, 0.41 mM MgSO_4_, 5.33 mM KCl, 0.44 mM KH_2_PO_4_, 4.17 mM NaHCO_3_, 137.93 mM NaCl, 0.34 mM Na_2_HPO_4_, 5.56 mM D-glucose, with or without 1.26 mM CaCl_2_) (Gibco-Thermo Fisher, MA, USA) and incubated for 10 min. Afterward, the cells were washed once with HBSS, and peptides were treated with HBSS. Time-lapse images were obtained using an Argon laser scanning confocal microscope (Leica TCS SP5 Microsystems, Wetzlar, Germany) at 10-s intervals for 10 min at an excitation of 488 nm for Fluo-4 and MitoSOX, or at 496 nm for DCF. As mitochondrial markers, MitoTracker Green (0.5 μM) and MitoTracker Red (0.1 μM) were preincubated (green for 30 min and red for 2 min), anti-TOMM20 antibody (Abcam, Cambridge, UK) was used for immunocytochemistry and immunofluorescence (ICC/IF), and mito-DsRed2 were transfected with Effectene (Qiagen, Hilden, Germany) on the day before confocal microscopy. Other details are available in the manufacturer’s instructions.

### Cobalt quenched calcein assay

HeLa cells were cultured on a Lab-Tek Chamber glass slide. Cells were incubated for 20 min in HBSS with Calcein-AM (1 μM) and Cobalt (CoCl_2_, 2 mM). Then, MitoTracker (0.1 μM in HBSS) was added and cells were incubated for 2 min. Before the treatment with peptides, cells were washed with HBSS once. Time-lapse images were obtained using an Argon laser scanning confocal microscope at intervals of 10 s for 10 min at 488 nm for Calcein-AM, and at 561 nm for MitoTracker.

### Two-step assay

DOPS, DOPE, and DOPC (Cat. #850375 P, 850725 P, and 840035 P, respectively; Avanti polar lipid, AL, USA) were dissolved in chloroform at a concentration of 5 mg/mL. PS:PC:PE = 2:4:3 were mixed in chloroform, and then chloroform was volatilized. The mixture was dissolved in TbCl_3_ buffer (15 mM TbCl_3_, 50 mM sodium citrate, 20 mM HEPES, and 150 mM NaCl (pH7.4)) at the concentration of 2 mg/mL for the capsulation of TbCl_3_. This solution was mixed using Mini extruder (Avanti polar lipid, AL, USA) with a 400 nm (400 kDa) filter for five times. The formed liposomes were washed with buffer (150 mM NaCl, 20 mM HEPES (pH7.4)) and spun down at 12,000 × *g* for 20 min for three times. Afterward, they were resuspended with assay buffer (50 μM DPA, 150 M NaCl, and 20 mM HEPES (pH7.4)) and 200 μL were added per well in a 96 well plate. Peptides were treated for 30 min and fluorescence was measured at 276 nm for excitation and at 490 nm for emission. All samples were analyzed in triplicate, and 0.1% triton was used as a positive control.

### Isolation of mitochondria

Mitochondria were isolated from livers of 6-week-old BalB/C mice. Mouse livers were minced and homogenized using the Teflon Potter-Elvehjem tissue grinder (Sigma-Aldrich, MO, USA) in washing buffer (250 mM mannitol, 70 mM sucrose, 0.5 mM EGTA, 5 mM HEPES, pH 7.4, 0.1 mM PMSF, and 4 μM rotenone). The homogenate was centrifuged at 1000 × *g* for 10 min at 4 °C and the supernatant was centrifuged at 10,000 × *g* for 10 min at 4 °C. The pellet was washed twice with regeneration buffer (250 mM sucrose, 10 mM HEPES, pH 7.4, 5 mM sodium succinate, 2 mM potassium phosphate, 0.1 mM PMSF, 25 μM EGTA, and 4 μM rotenone). The pellet was resuspended in regeneration buffer for the subsequent experiments.

### Super-resolution microscopy

PALM imaging was performed using ELYRA P.1 microscope system (ZIESS, Oberkochen, Germany)^37^ at Korea Basic Science Institute, Gwangju branch. HeLa cells were cultured on glass bottom dishes (Greiner, Frickenhausen, Germany) for 60–70% of confluency, and the vectors were transfected into HeLa cells using Effectene (Qiagen, Hilden, Germany). After 24 h, cells were washed once with prewarmed (37 °C) HBSS buffer, and fixed immediately with prewarmed 2% paraformaldehyde for 15 min at 37 °C. The fixed cells were washed with glycine 100 mM in PHEM buffer (60 mM PIPES, 25 mM HEPES, 10 mM EGTA, 2 mM MgCl_2_, and pH 6.9) to stop the fixation reaction, and washed with PHEM buffer for three times. A total of 405 and 488 nm lasers were used for the activation of rsKame, and 405 and 561 nm lasers were used for the activation of PAmCherry.

### Atomic forced microscopy

MDA-MB-231 cells were plated on well plates and treated with eMTD∆4 for the indicated time. Then, the cells were fixed with 4% paraformaldehyde. Images of the cell surface were obtained using an atomic force microscopy (NANO Station II, Herzogenrath, Germany)^38,39^. All images were obtained in non-contact mode with a resolution of 512 × 512 pixels and a scan speed of 0.2 lines/s. The specifications of cantilever with a pyramidal-shaped tip used in this work were as follows: a frequency of 146–236 kHz, a spring constant of 21–98 N/m, a length of 225 nm, and a resistance of 0.01–0.02 Ω cm. The images were analyzed with SPIP software (Scanning Probe Image Processor, v4.1; Image Metrology, Horsholm, Denmark).

## Supplementary information

Extended figure 1

Extended figure 2

Extended figure 3

Extended figure 4

Extended figure 5

Supplementary figure legends

## References

[1] Guikema JE, Amiot M, Eldering E (2017). Exploiting the pro-apoptotic function of NOXA as a therapeutic modality in cancer. Expert Opin. therapeutic Targets.

[2] Lopez H (2010). Perturbation of the Bcl-2 network and an induced Noxa/Bcl-xL interaction trigger mitochondrial dysfunction after DNA damage. J. Biol. Chem..

[3] Chen L (2005). Differential targeting of prosurvival Bcl-2 proteins by their BH3-only ligands allows complementary apoptotic function. Mol. Cell.

[4] Willis SN (2005). Proapoptotic Bak is sequestered by Mcl-1 and Bcl-xL, but not Bcl-2, until displaced by BH3-only proteins. Genes Dev..

[5] Seo YW (2003). The molecular mechanism of Noxa-induced mitochondrial dysfunction in p53-mediated cell death. J. Biol. Chem..

[6] Woo HN (2009). Effects of the BH3-only protein human Noxa on mitochondrial dynamics. FEBS Lett..

[7] Seo YW (2009). The cell death-inducing activity of the peptide containing Noxa mitochondrial-targeting domain is associated with calcium release. Cancer Res..

[8] Kim JY (2013). Minimal killing unit of the mitochondrial targeting domain of Noxa. J. Pept. Sci. Off. Publ. Eur. Pept. Soc..

[9] Ponnalagu D, Singh H (2017). Anion Channels of Mitochondria. Handb. Exp. Pharmacol..

[10] Naghdi S, Hajnoczky G (2016). VDAC2-specific cellular functions and the underlying structure. Biochim. Biophys. Acta.

[11] Cheng EH, Sheiko TV, Fisher JK, Craigen WJ, Korsmeyer SJ (2003). VDAC2 inhibits BAK activation and mitochondrial apoptosis. Science.

[12] Ma SB (2014). Bax targets mitochondria by distinct mechanisms before or during apoptotic cell death: a requirement for VDAC2 or Bak for efficient Bax apoptotic function. Cell Death Differ..

[13] Shimizu S, Ide T, Yanagida T, Tsujimoto Y (2000). Electrophysiological study of a novel large pore formed by Bax and the voltage-dependent anion channel that is permeable to cytochrome c. J. Biol. Chem..

[14] Tedeschi H, Harris DL (1955). The osmotic behavior and permeability to non-electrolytes of mitochondria. Arch. Biochem. Biophys..

[15] Woodfield K, Ruck A, Brdiczka D, Halestrap AP (1998). Direct demonstration of a specific interaction between cyclophilin-D and the adenine nucleotide translocase confirms their role in the mitochondrial permeability transition. Biochem. J..

[16] Petronilli V (1998). Imaging the mitochondrial permeability transition pore in intact cells. BioFactors.

[17] Maurya SR, Mahalakshmi R (2016). VDAC-2: Mitochondrial outer membrane regulator masquerading as a channel?. FEBS J..

[18] De Pinto V, Reina S, Gupta A, Messina A, Mahalakshmi R (2016). Role of cysteines in mammalian VDAC isoforms’ function. Biochim. Biophys. Acta.

[19] Lauterwasser J (2016). The porin VDAC2 is the mitochondrial platform for Bax retrotranslocation. Sci. Rep..

[20] Keinan N, Tyomkin D, Shoshan-Barmatz V (2010). Oligomerization of the mitochondrial protein voltage-dependent anion channel is coupled to the induction of apoptosis. Mol. Cell. Biol..

[21] Nakagawa T (2005). Cyclophilin D-dependent mitochondrial permeability transition regulates some necrotic but not apoptotic cell death. Nature.

[22] Ding J (2016). Pore-forming activity and structural autoinhibition of the gasdermin family. Nature.

[23] Oda E (2000). Noxa, a BH3-only member of the Bcl-2 family and candidate mediator of p53-induced apoptosis. Science.

[24] Lorents A (2012). Cell-penetrating peptides split into two groups based on modulation of intracellular calcium concentration. J. Biol. Chem..

[25] Roos WP, Kaina B (2006). DNA damage-induced cell death by apoptosis. Trends Mol. Med..

[26] Vandenberghe I (2008). Physalin B, a novel inhibitor of the ubiquitin-proteasome pathway, triggers NOXA-associated apoptosis. Biochem. Pharmacol..

[27] Nys K (2012). Skin mild hypoxia enhances killing of UVB-damaged keratinocytes through reactive oxygen species-mediated apoptosis requiring Noxa and Bim. Free Radic. Biol. Med..

[28] Kim J-Y, Ahn H-J, Ryu J-H, Suk K, Park J-H (2004). BH3-only protein noxa is a mediator of hypoxic cell death induced by hypoxia-inducible factor 1α. J. Exp. Med..

[29] Kalogeris T, Baines CP, Krenz M, Korthuis RJ (2012). Cell biology of ischemia/reperfusion. Injury. Int. Rev. Cell Mol. Biol..

[30] Wang X (2015). DIDS reduces ischemia/reperfusion-induced myocardial injury in rats. Cell. Physiol. Biochem..

[31] Mazure NM (2017). VDAC in cancer. Biochim. Biophys. Acta. Bioenerg..

[32] De Pinto V, Messina A, Lane DJ, Lawen A (2010). Voltage-dependent anion-selective channel (VDAC) in the plasma membrane. FEBS Lett..

[33] Draeger A, Schoenauer R, Atanassoff AP, Wolfmeier H, Babiychuk EB (2014). Dealing with damage: plasma membrane repair mechanisms. Biochimie.

[34] Koch GL (1990). The endoplasmic reticulum and calcium storage. BioEssays.

[35] Contreras L, Drago I, Zampese E, Pozzan T (2010). Mitochondria: the calcium connection. Biochim. Biophys. Acta Bioenerg..

[36] Bagur R, Hajnoczky G (2017). Intracellular Ca(2+) sensing: its role in calcium homeostasis and signaling. Mol. Cell.

[37] Annibale P, Vanni S, Scarselli M, Rothlisberger U, Radenovic A (2011). Quantitative photo activated localization microscopy: unraveling the effects of photoblinking. PloS ONE.

[38] Eghiaian F, Rico F, Colom A, Casuso I, Scheuring S (2014). High-speed atomic force microscopy: imaging and force spectroscopy. FEBS Lett..

[39] Churnside AB, Perkins TT (2014). Ultrastable atomic force microscopy: improved force and positional stability. FEBS Lett..

