# Supplementary figures and images for: Noxa mitochondrial targeting domain induces necrosis via VDAC2 and mitochondrial catastrophe
Source: Cell Death Dis. 2019 Jul 8;10(7):519. doi: 10.1038/s41419-019-1753-4 (PMC6614423; doi:10.1038/s41419-019-1753-4)

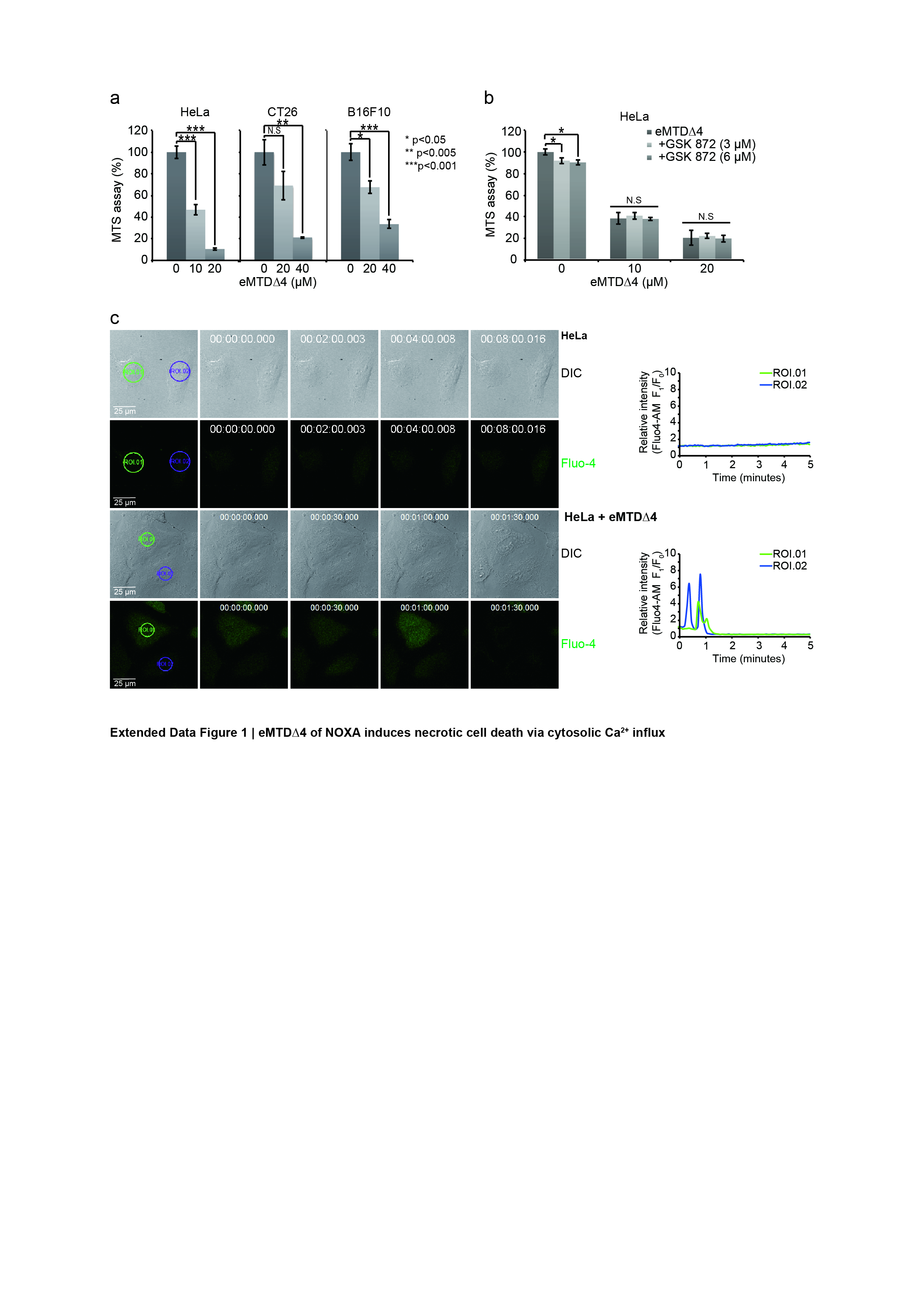

Supplement: Supplementary file 1 — Extended figure 1 [file 41419_2019_1753_MOESM1_ESM.tif]

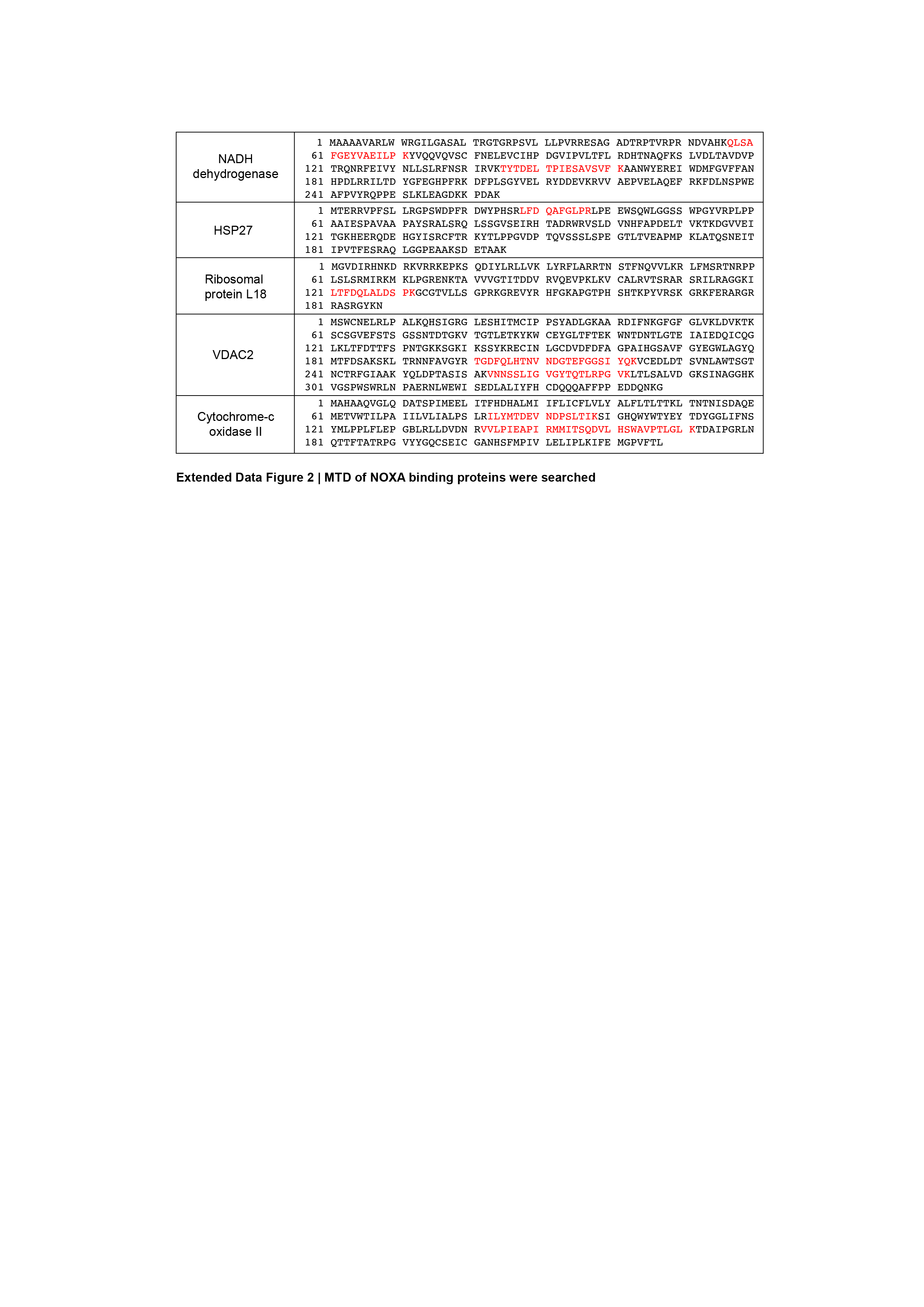

Supplement: Supplementary file 2 — Extended figure 2 [file 41419_2019_1753_MOESM2_ESM.tif]

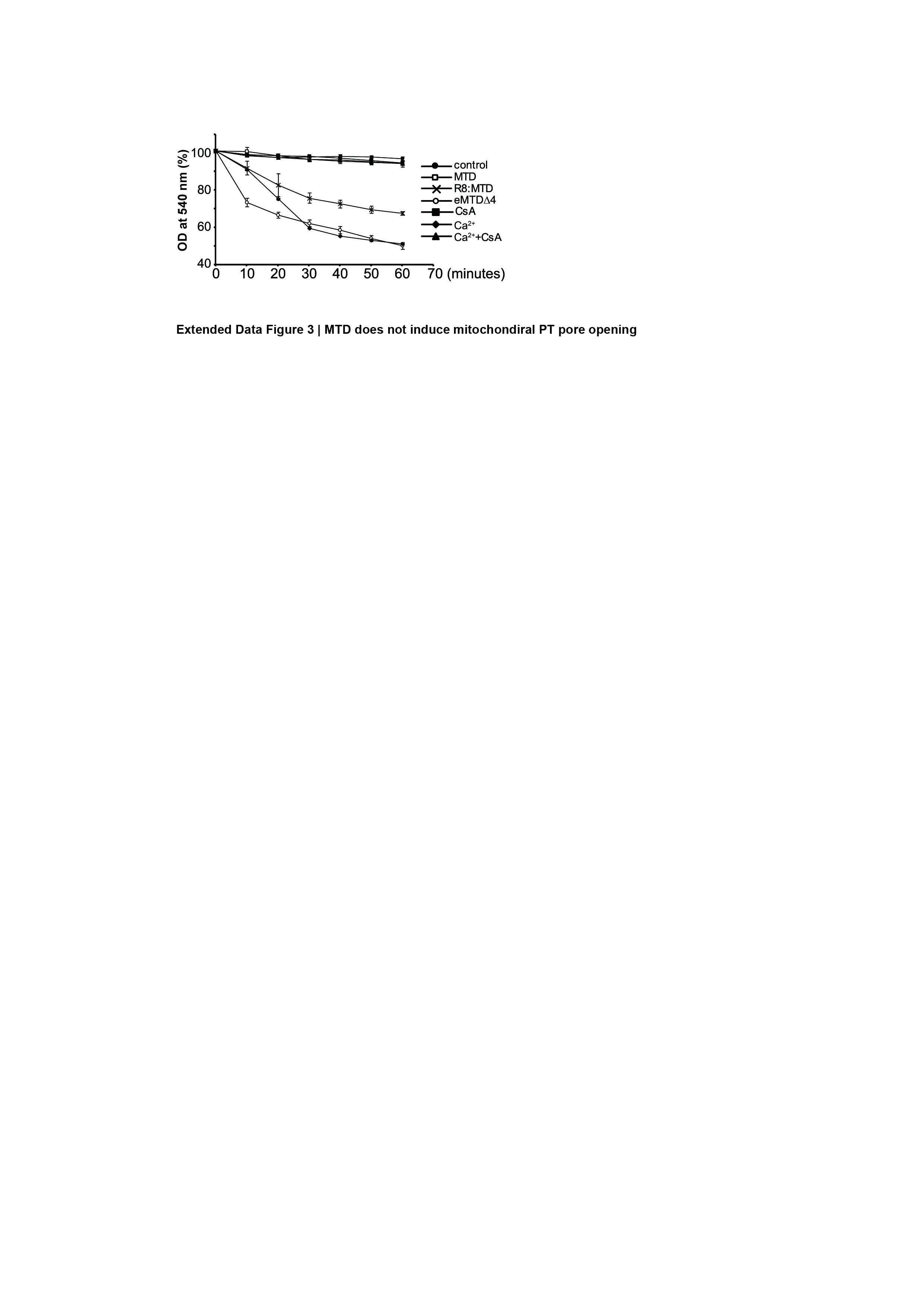

Supplement: Supplementary file 3 — Extended figure 3 [file 41419_2019_1753_MOESM3_ESM.tif]

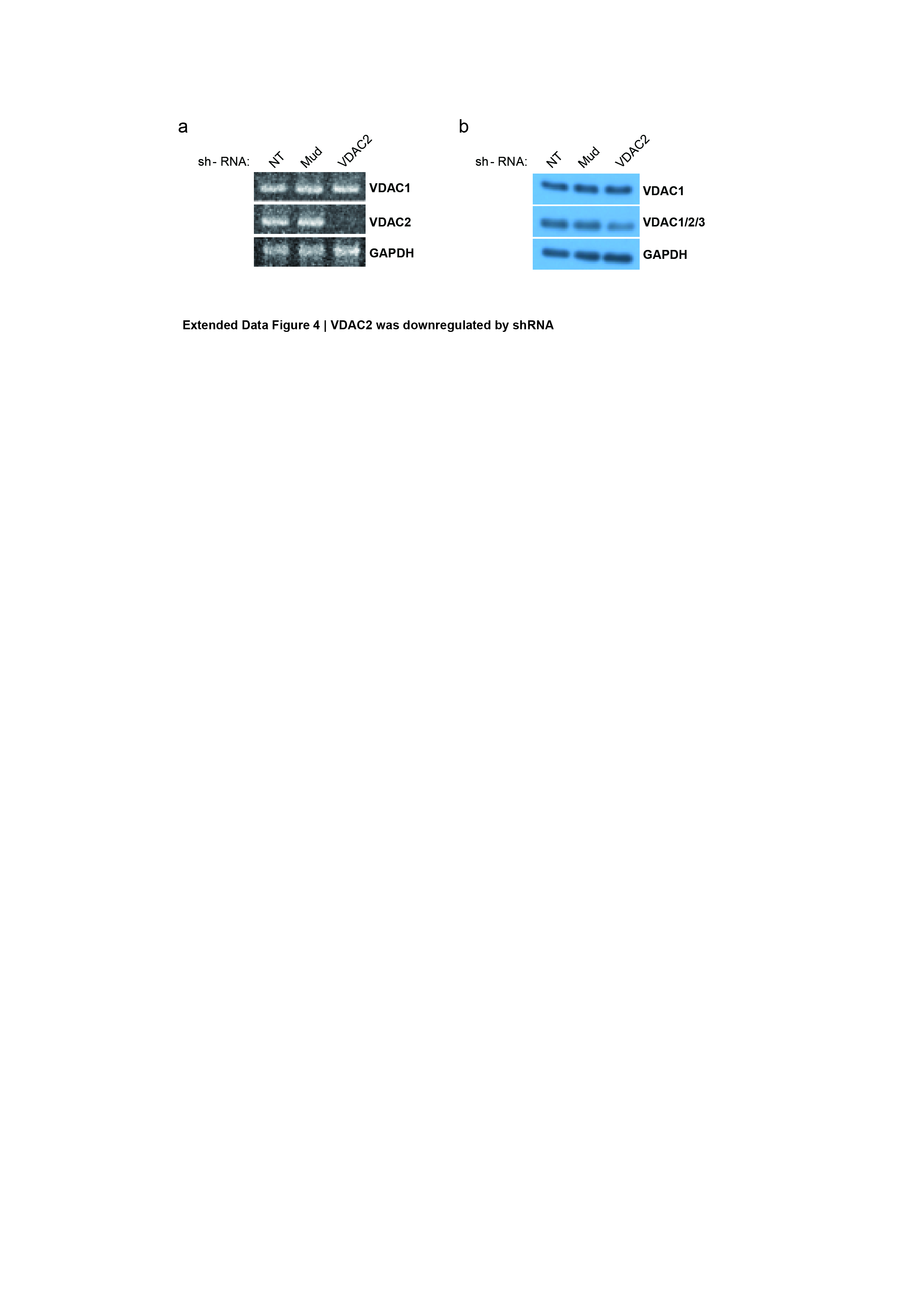

Supplement: Supplementary file 4 — Extended figure 4 [file 41419_2019_1753_MOESM4_ESM.tif]

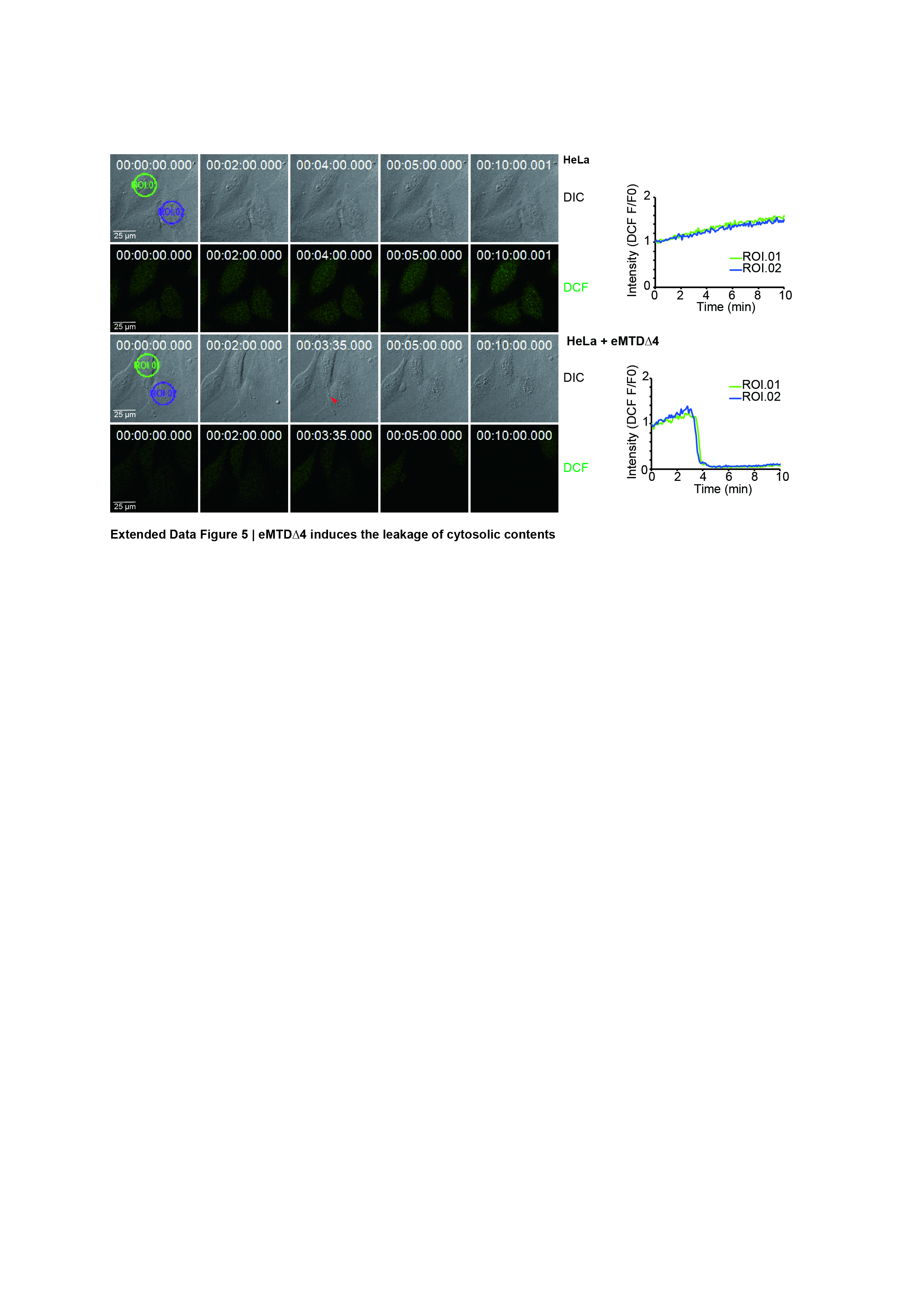

Supplement: Supplementary file 5 — Extended figure 5 [file 41419_2019_1753_MOESM5_ESM.tif]
