# Supplementary material for: Noxa mitochondrial targeting domain induces necrosis via VDAC2 and mitochondrial catastrophe
Source: Cell Death Dis. 2019 Jul 8;10(7):519. doi: 10.1038/s41419-019-1753-4 (PMC6614423; doi:10.1038/s41419-019-1753-4)
Supplement: Supplementary file 6 — Supplementary figure legends [file 41419_2019_1753_MOESM6_ESM.zip]

**Extended Data** **Figure 1 | eMTDΔ4 of NOXA induces necrotic cell death via cytosolic Ca^2+^ influx.** a and b, HeLa, CT26 and B16F10 were treated with eMTDΔ4 (a). HeLa cells were treated with eMTDΔ4 and RIP3 inhibitor GSK 872 (b). Cell viability was determined by the MTS assay. c, Images of HeLa cells with or without eMTDΔ4 treatment in HBSS buffer without Ca^2+^ were captured by time-lapse confocal microscopy. Cytosolic Ca^2+^ concentration was monitored using Fluo-4-AM. The relative fluorescent intensity of Fluo-4-AM in two regions of interest (ROI) was graphed over time.

**Extended Data** **Figure 2 | MTD of NOXA binding proteins were searched.** Several candidates were identified by MicroQ-TOF III mass spectrometer, and the sequences identified were shown in red.

**Extended Data** **Figure 3 | MTD does not induce mitochondrial PT pore opening.** Isolated mitochondria from BalB/c mouse liver were treated with MTD (50 μM), R8:MTD (50 μM), and eMTDΔ4 (25 μM), CsA (20 μM), Ca^2+^ (200 μM), and Ca^2+^ (200 μM) plus CsA (20 μM), and OD at 540 nm was monitored.

**Extended Data** **Figure 4 | VDAC2 was downregulated by shRNA**

a and b, HeLa cells were transfected with shRNA expression vectors (sh-NT for no target gene, sh-Mud for MUDENG, sh-VDAC2 for VDAC2). mRNA level (a) and expression level (b) were observed.

**Extended Data** **Figure 5 | eMTDΔ4 induces the leakage of cytosolic contents.** HeLa cells were treated with eMTDΔ4 after incubation with the cytosolic ROS indicator DCF. The images were taken by time-lapse confocal microscope. The relative fluorescence intensity in two regions of interest were calculated over time.
